# Contrast-enhanced ultrasound enhancement-pattern classification for procedure selection and perioperative outcomes in cesarean scar pregnancy

**DOI:** 10.3389/fsurg.2026.1818213

**Published:** 2026-05-14

**Authors:** Jinfeng Yang, Zhongya Xu, Xueke Liu, Jin Feng, Ying Niu

**Affiliations:** Department of Ultrasound, The People's Hospital of Yingshang, Yingshang, China

**Keywords:** cesarean scar pregnancy, contrast-enhanced ultrasound, enhancement pattern, procedure selection, transvaginal ultrasound

## Abstract

**Objective:**

To develop a pragmatic contrast-enhanced ultrasound (CEUS) enhancement-pattern classification for cesarean scar pregnancy (CSP) and evaluate its association with procedure selection and perioperative outcomes.

**Methods:**

We retrospectively analyzed consecutive CSP patients who underwent pre-procedural CEUS at The People's Hospital of Yingshang (Yingshang County, Anhui, China) between August 2021 and January 2026. In our center, once outpatient two-dimensional ultrasound raised suspicion for CSP and the patient was admitted, CEUS was routinely performed as part of the preoperative assessment to evaluate the relationship between the scar lesion and contrast enhancement morphology. CSP severity was recorded using the 2016 transvaginal ultrasound (TVS) Type I-III system. CEUS patterns were classified into five predefined subtypes (ICSPF, ICSPB, CCSPF, CCSPB, and MA) according to early wash-in distribution, extent of enhancement, and vascular architecture, and then linked to a three-tier procedure recommendation. Two experienced ultrasound physicians reviewed representative cases after standardized training, showing substantial interobserver agreement for the TVS classification (*κ* = 0.775) and the CEUS enhancement-pattern classification (*κ* = 0.800). Concordance between CEUS-based recommendation and definitive procedure was assessed using Cohen's kappa. Perioperative outcomes included operative time, estimated blood loss (EBL), length of stay, and adverse events. Incremental value beyond TVS was assessed using logistic regression for predicting lesion resection plus scar repair.

**Results:**

Among 131 patients, CEUS patterns were strongly associated with conventional ultrasound severity markers, including TVS type, residual myometrial thickness, and scar thickness. CEUS-based recommendations showed 90.1% concordance with definitive management (*κ* = 0.82). Perioperative outcomes differed by procedure, with lesion resection plus scar repair associated with greater procedural burden. Adding a CEUS high-risk pattern (CCSPB) improved prediction of cases managed with scar repair compared with a TVS-based model.

**Conclusion:**

CEUS enhancement-pattern classification aligns with established ultrasound severity indicators and supports real-world procedure selection in CSP. The CCSPB pattern may provide incremental value beyond selected TVS parameters for identifying patients who ultimately require scar repair, although this finding should be interpreted cautiously. Prospective multicenter validation is warranted.

## 1 Introduction

Cesarean scar pregnancy (CSP) is an uncommon but potentially life-threatening form of ectopic implantation within the myometrial defect of a prior cesarean section scar (1, 2). With the rising global cesarean delivery rate and improved first-trimester imaging, reported CSP diagnoses have increased, and the condition is recognized as a major contributor to severe hemorrhage, uterine rupture, and loss of fertility if not managed appropriately (1–3).

Beyond acute complications, accumulating evidence supports a pathophysiologic continuum between CSP and placenta accreta spectrum, with shared histopathology and abnormal trophoblast invasion at the scar niche (4). Accordingly, early standardized ultrasound assessment is central to risk stratification and counseling. Recent efforts have proposed consensus definitions and structured sonographic reporting elements (including implantation site, growth pattern, and residual myometrial thickness), improving inter-study comparability and supporting individualized management (5, 6).

**Figure 1:**
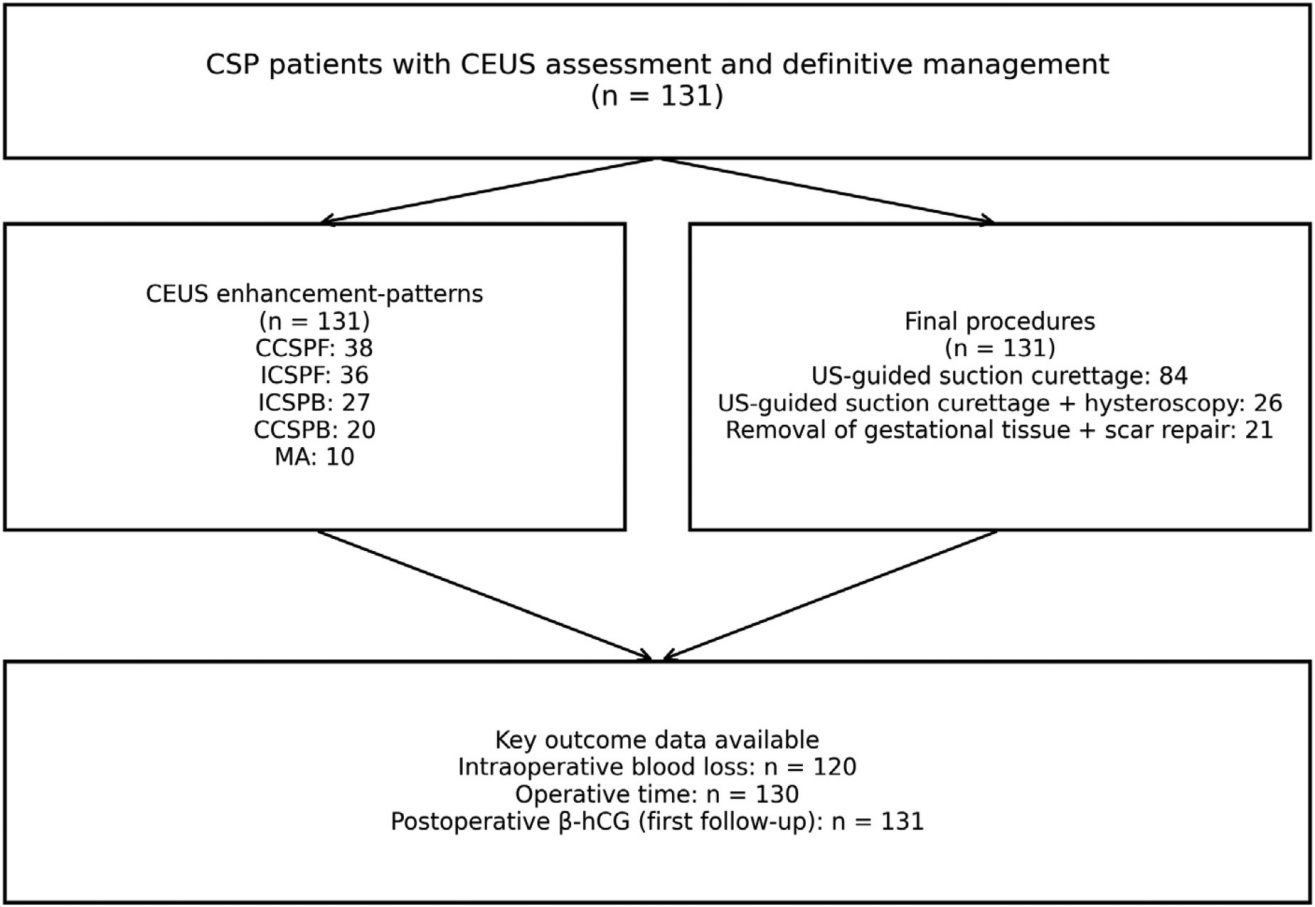
Study flow and data availability. The cohort included 131 CSP patients with CEUS assessment and definitive management. CEUS enhancement-pattern categories and final procedures are summarized, and availability of key perioperative/outcome variables is shown.

Despite increasing guidance, CSP management remains heterogeneous, ranging from minimally invasive uterine aspiration or hysteroscopic/laparoscopic resection to combined embolization and surgical approaches, with treatment choice strongly influenced by bleeding risk, myometrial thickness, vascularity, and operator expertise (7–9). Large observational series and contemporary evidence syntheses highlight substantial variation in perioperative outcomes across modalities, underscoring the need for practical imaging-driven decision support tools (10, 11). The study flow and availability of key outcome data are summarized in Figure 1.

Contrast-enhanced ultrasound (CEUS) can depict microvascular perfusion and scar-associated neovascularization beyond conventional color Doppler (12). Prior studies have suggested that CEUS may improve diagnostic confidence and characterization of cesarean scar pregnancy compared with transvaginal ultrasound alone (13, 14). CEUS has also been explored to assist in the evaluation of interventional management, including uterine artery embolization (15). However, existing CEUS work in CSP has largely emphasized diagnosis or broad typing, with limited integration of enhancement patterns into explicit procedure-selection pathways and perioperative outcome prediction. Therefore, we aimed to develop a pragmatic CEUS enhancement-pattern classification and evaluate its association with procedure selection and perioperative outcomes in a real-world CSP cohort.

## 2 Materials and methods

### 2.1 Study design and setting

This retrospective observational study was conducted at the Department of Ultrasound, The People's Hospital of Yingshang (Yingshang County, Anhui, China). Consecutive patients diagnosed with cesarean scar pregnancy (CSP) who underwent contrast-enhanced ultrasound (CEUS) prior to interventional management between 12-Aug-2021 and 13-Jan-2026 were screened for eligibility. During the study period, once outpatient two-dimensional ultrasound raised suspicion for CSP and the patient was admitted, CEUS was routinely incorporated into the institutional pre-procedural workflow for preoperative assessment of lesion-scar enhancement morphology. Therefore, the analytic cohort reflects the local consecutive inpatient CEUS workflow rather than a selectively CEUS-enriched subgroup. The study was reported in accordance with the STROBE guideline for observational studies.

Ethics approval: The study protocol was approved by the Institutional Review Board of The People's Hospital of Yingshang (Approval No. [2020] 18). Given the retrospective design and use of de-identified data, the requirement for informed consent was waived in accordance with local regulations.

### 2.2 Participants

Inclusion criteria were: (1) positive serum beta-human chorionic gonadotropin (beta-hCG) consistent with early pregnancy; (2) a history of at least one cesarean delivery; (3) CSP diagnosis on transvaginal ultrasound (TVS) based on accepted sonographic criteria; and (4) pre-procedural CEUS available for classification. Patients were excluded if they had contraindications to ultrasound contrast agents, incomplete imaging precluding classification, or incomplete key perioperative records. At our institution, once outpatient two-dimensional ultrasound raised suspicion for CSP and the patient was admitted, CEUS was routinely incorporated into the preoperative inpatient assessment; therefore, the analytic cohort approximates a consecutive CEUS-assessed inpatient CSP series within this local workflow.

### 2.3 Ultrasound and CEUS protocol

All patients underwent TVS using a GE LOGIQ E9 system with a transvaginal probe (5.0–7.5 MHz). Standard grayscale and color Doppler images were acquired to document lesion location, maximal gestational sac diameter, embryo viability (if present), scar thickness, and residual myometrial thickness (RMT). In our routine workflow, patients with suspected CSP on outpatient two-dimensional ultrasound underwent CEUS after admission as part of preoperative assessment. CEUS was performed immediately after conventional ultrasound using a low mechanical index contrast-specific mode (typical MI 0.05–0.08). A second-generation microbubble contrast agent (Sonazoid, 0.015 mL/kg) was administered as a bolus via a peripheral vein followed by a saline flush (5 mL). Continuous cine loops were recorded for at least 3 min to capture wash-in and wash-out, with imaging planes centered on the implantation site and adjacent myometrium. Pattern assignment focused primarily on early wash-in behavior and considered enhancement location, depth, circumferential extent, continuity across the scar thickness, and the presence of dense or disorganized vascular architecture.

### 2.4 Imaging classifications

CSP was categorized on TVS using the 2016 ultrasound-based Type I-III classification recorded in the clinical database. In parallel, conventional sonographic severity markers relevant to current CSP stratification - including TVS type, residual myometrial thickness, scar thickness, and embryo viability - were abstracted for analysis. Because this was a retrospective database-based study, other descriptors increasingly used in contemporary CSP frameworks, such as growth direction, crossover sign, and formal vascularity grading, were not uniformly archived for every case and therefore were not entered as core analytic variables.

CEUS enhancement patterns were classified *a priori* using early wash-in cine loops, with reference to enhancement distribution within the scar niche, extent of involvement across the implantation bed, continuity toward the serosal/bladder side, and vascular architecture. ICSPF was defined as a relatively focal enhancement pattern confined to a limited portion of the scar implantation zone without broad trans-scar extension. ICSPB was defined as a broader inward/superficial enhancement pattern involving a larger segment of the niche but without diffuse full-thickness confluent enhancement. CCSPF was defined as a deeper or more continuous enhancement pattern traversing the scar implantation site while remaining relatively localized in overall extent. CCSPB was defined as broad, confluent enhancement involving most or all of the implantation bed, typically extending across the scar thickness toward the serosal/bladder side and accompanied by dense vascular architecture. MA was defined as an irregular mass-like and heterogeneous enhancement pattern with disorganized vessels and loss of an orderly layered appearance. When mixed features were present, the dominant or highest-risk early wash-in component was used for final categorization.

To improve classification consistency, two experienced ultrasound physicians underwent unified training on the predefined TVS and CEUS classification criteria and independently reviewed representative cases for agreement assessment. Interobserver agreement was substantial for the 2016 TVS classification (*κ* = 0.775) and for the CEUS enhancement-pattern classification (*κ* = 0.800). In the full retrospective cohort, the final CEUS category used for analysis was the prospectively recorded clinical category assigned before definitive treatment. Because the study was retrospective and not designed as a fully blinded reading trial, complete blinding to clinical context was not ensured, and this issue is acknowledged in the limitations.

An additional binary flag (“guideline repair recommended”) recorded in the clinical database indicated whether scar repair was recommended under the institutional CSP management algorithm in place during the study period.

### 2.5 CEUS-guided procedure recommendation and interventions

Based on the CEUS enhancement-pattern subtype, a pre-procedural recommendation was recorded for one of the following strategies: (1) ultrasound-guided dilation and curettage (D&C); (2) ultrasound-guided D&C plus hysteroscopy; or (3) lesion resection plus scar repair (laparoscopic, transvaginal, or combined according to surgical judgment). This recommendation reflected the local real-world management pathway and was used in the present study to assess whether the CEUS classification aligned with definitive treatment selection, rather than to imply that CEUS alone determined the final procedure.

### 2.6 Outcomes

The primary perioperative outcomes were operative time (min), estimated blood loss (EBL, mL), and length of stay (LOS, days). Clinical adverse events were abstracted from operative and inpatient records as coded in the database. Clinically significant bleeding was additionally described using an EBL threshold of >200 mL among cases with EBL recorded. Early postoperative beta-hCG values were incompletely available in routine follow-up and were therefore analyzed on an available-case basis only; these data were treated as descriptive secondary information rather than a primary comparative endpoint.

### 2.7 Statistical analysis

All analyses were performed using IBM SPSS Statistics (version 31.0.1.0, IBM Corp., Armonk, NY, USA). Continuous variables are presented as mean ± standard deviation (SD) if normally distributed, or median (interquartile range, IQR) otherwise. Between-group comparisons were performed using one-way ANOVA or Kruskal–Wallis tests for continuous variables and chi-square or Fisher's exact tests for categorical variables, as appropriate. Concordance between CEUS-based recommendation and definitive management was quantified using Cohen's kappa. To explore incremental value beyond TVS, multivariable logistic regression was used to model lesion resection plus scar repair, with effect estimates reported as odds ratios (ORs) and 95% confidence intervals (CIs). Given the limited number of scar-repair events, the regression analysis was considered exploratory; formal internal validation and model-calibration analyses were not undertaken.

## 3 Results

### 3.1 Study population and baseline characteristics

A total of 131 patients with cesarean scar pregnancy (CSP) were included between 12-Aug-2021 and 13-Jan-2026 (index date missing in 2 cases). In this center, CEUS was routinely performed after admission when outpatient two-dimensional ultrasound suggested CSP, so the cohort reflects the local consecutive inpatient CEUS workflow rather than a selectively CEUS-enriched subgroup. The median age was 34.0 years (IQR 31.0–37.0), and the median gestational age at presentation was 6.57 weeks (IQR 6.00–7.57). The median number of prior cesarean deliveries was 2 (IQR 2–3), median maximal gestational sac diameter was 27.0 mm (IQR 20.0–36.0), and median scar thickness was 2.8 mm (IQR 2.0–3.8). Residual myometrial thickness of <=3 mm was present in 71/131 (54.2%), a viable embryo in 61/131 (46.6%), and guideline repair recommendation in 69/131 (52.7%). By TVS2016 classification, 62/131 (47.3%) were Type I, 44/131 (33.6%) Type II, and 25/131 (19.1%) Type III (Table 1).

**Table 1:** Baseline characteristics of the cohort.

Characteristic	Overall (*N* = 131)
Age, years	34.0 (31.0–37.0)
Gestational age, weeks	6.57 (6.00–7.57)
Prior cesarean deliveries, *n*	2 (2–3)
Max gestational sac diameter, mm	27.0 (20.0–36.0)
Scar thickness, mm	2.8 (2.0–3.8)
RMT ≤3 mm	71/131 (54.2%)
Viable embryo	61/131 (46.6%)
TVS2016 classification: Type I	62/131 (47.3%)
TVS2016 classification: Type II	44/131 (33.6%)
TVS2016 classification: Type III	25/131 (19.1%)
Guideline repair recommended	69/131 (52.7%)

Values are median (IQR) or *n*/*N* (%).

### 3.2 CEUS enhancement-pattern classification

**Figure 2:**
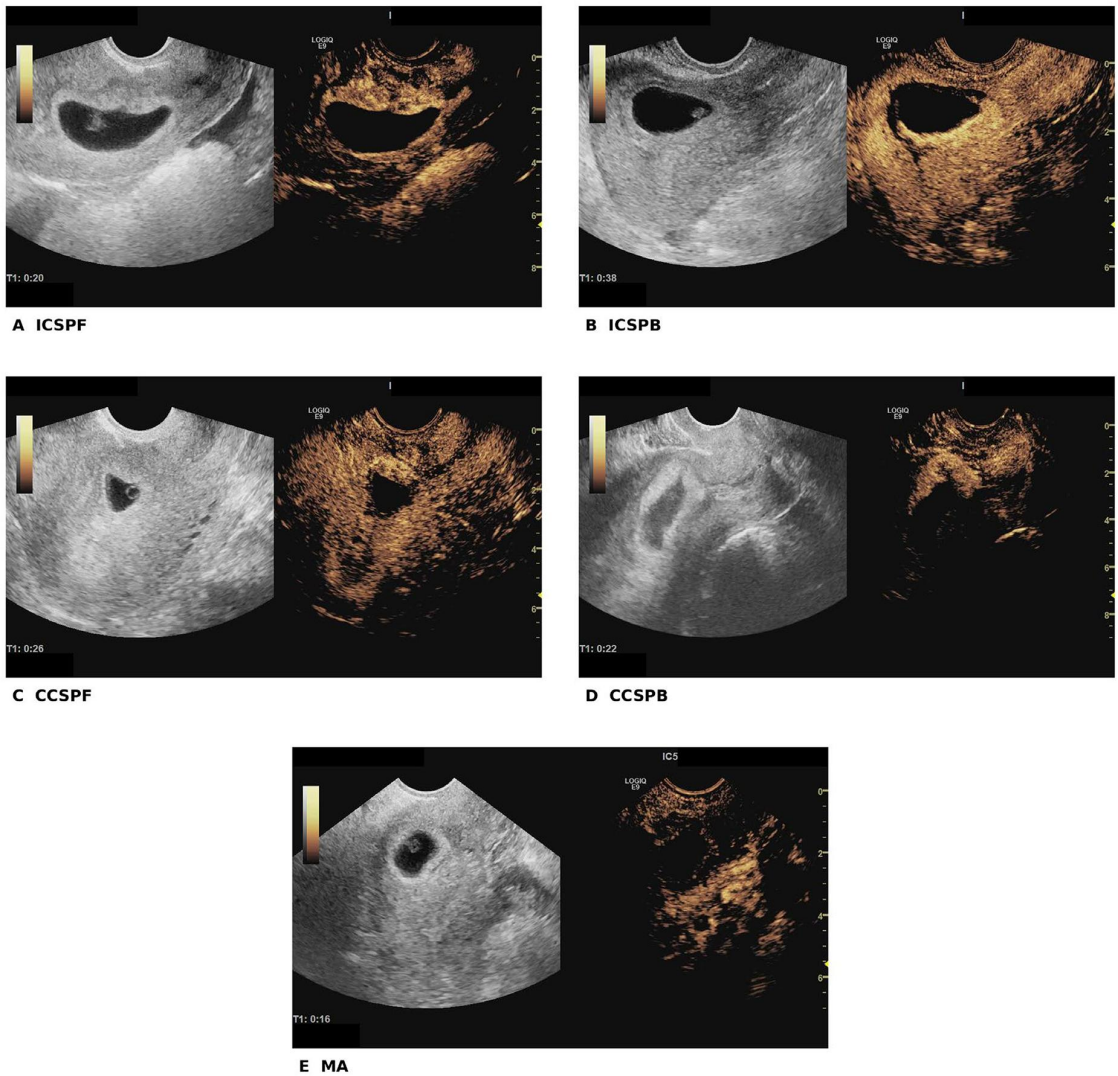
Representative CEUS images of the five enhancement-pattern subtypes in cesarean scar pregnancy. **(A)** ICSPF; **(B)** ICSPB; **(C)** CCSPF; **(D)** CCSPB; and **(E)** MA. In each panel, the grayscale transvaginal ultrasound image is shown on the left and the corresponding contrast-enhanced ultrasound image is shown on the right.

**Figure 3:**
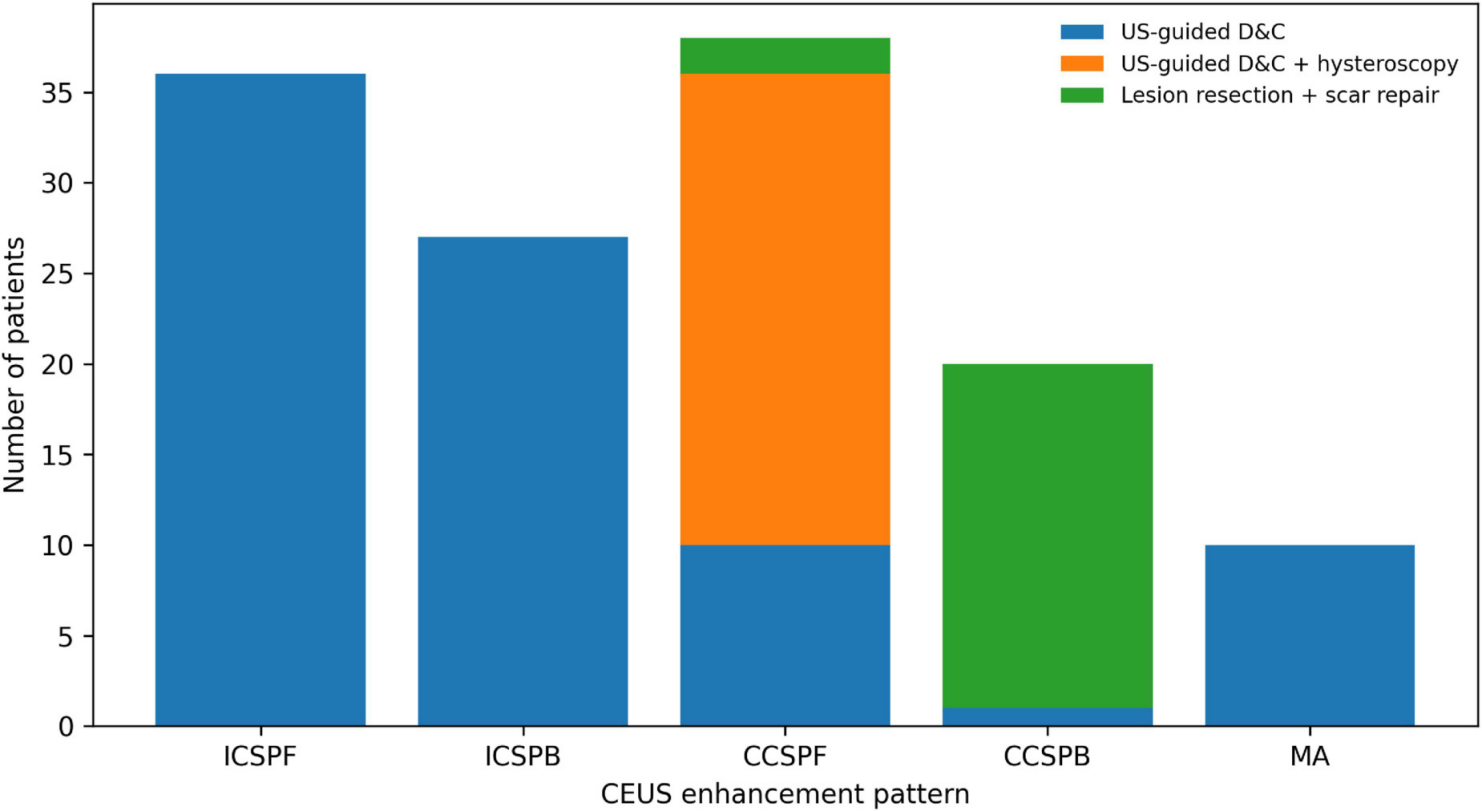
Definitive procedure distribution by CEUS enhancement pattern.

CEUS enhancement-pattern classification yielded 36 (27.5%) ICSPF, 27 (20.6%) ICSPB, 38 (29.0%) CCSPF, 20 (15.3%) CCSPB, and 10 (7.6%) MA patterns. Representative CEUS images of the five enhancement-pattern subtypes are shown in Figure 2. In the additional agreement assessment, interobserver consistency was substantial for both the 2016 TVS classification (*κ* = 0.775) and the CEUS enhancement-pattern classification (*κ* = 0.800). CEUS patterns were strongly associated with TVS2016 type (chi-square *P* < 0.001). Increasing anatomic severity was observed across patterns: the proportion of RMT ≤3 mm rose from 25.0% in ICSPF to 95.0% in CCSPB (chi-square *P* < 0.001), whereas median scar thickness decreased from 3.7 mm (ICSPF) to 1.8 mm (CCSPB) (Kruskal–Wallis *P* < 0.001). Pattern-level characteristics and downstream procedures are summarized in Table 2. Definitive procedure selection across CEUS patterns is visualized in Figure 3.

**Table 2:** CEUS pattern distribution, imaging severity markers, and definitive procedures.

CEUS pattern	*N*	TVS type I/II/III (*n*)	RMT ≤3 mm	Scar thickness, mm	Viable embryo	Guideline repair recommended	Final procedure: D&C/D&C + Hys/Repair (*n*)
ICSPF	36	28/8/0	9/36 (25.0%)	3.7 (3.1–5.6)	22/36 (61.1%)	8/36 (22.2%)	36/0/0
ICSPB	27	16/10/1	13/27 (48.1%)	3.0 (2.5–4.5)	19/27 (70.4%)	11/27 (40.7%)	27/0/0
CCSPF	38	15/18/5	23/38 (60.5%)	2.5 (2.0–3.4)	13/38 (34.2%)	23/38 (60.5%)	10/26/2
CCSPB	20	0/2/18	19/20 (95.0%)	1.8 (1.1–2.2)	6/20 (30.0%)	20/20 (100.0%)	1/0/19
MA	10	3/6/1	7/10 (70.0%)	2.8 (2.1–2.9)	1/10 (10.0%)	7/10 (70.0%)	10/0/0

TVS type is shown as Type I/Type II/Type III counts. Definitive procedures are shown as D&C/D&C + hysteroscopy/lesion resection+scar repair.

### 3.3 CEUS-guided procedure recommendation and concordance

According to CEUS-based recommendations recorded in the dataset, 73/131 (55.7%) patients were triaged to ultrasound-guided dilation and curettage (D&C), 36/131 (27.5%) to ultrasound-guided D&C plus hysteroscopy, and 22/131 (16.8%) to lesion resection plus scar repair. The final procedures performed were 84/131 (64.1%) D&C, 26/131 (19.8%) D&C plus hysteroscopy, and 21/131 (16.0%) lesion resection plus scar repair. Overall, final management was concordant with the CEUS-based recommendation in 118/131 (90.1%) cases (Cohen's *κ* = 0.82). The cross-tabulation is shown in Table 3.

**Table 3:** CEUS-based recommendation vs. definitive procedure performed.

Recommended procedure	Final: US-guided D&C	Final: US-guided D&C + hysteroscopy	Final: Lesion resection+scar repair
Recommended: US-guided D&C	73	0	0
Recommended: US-guided D&C + hysteroscopy	10	25	1
Recommended: Lesion resection+scar repair	1	1	20

Overall concordance 118/131 (90.1%); Cohen's kappa=0.82.

### 3.4 Perioperative outcomes

**Figure 4:**
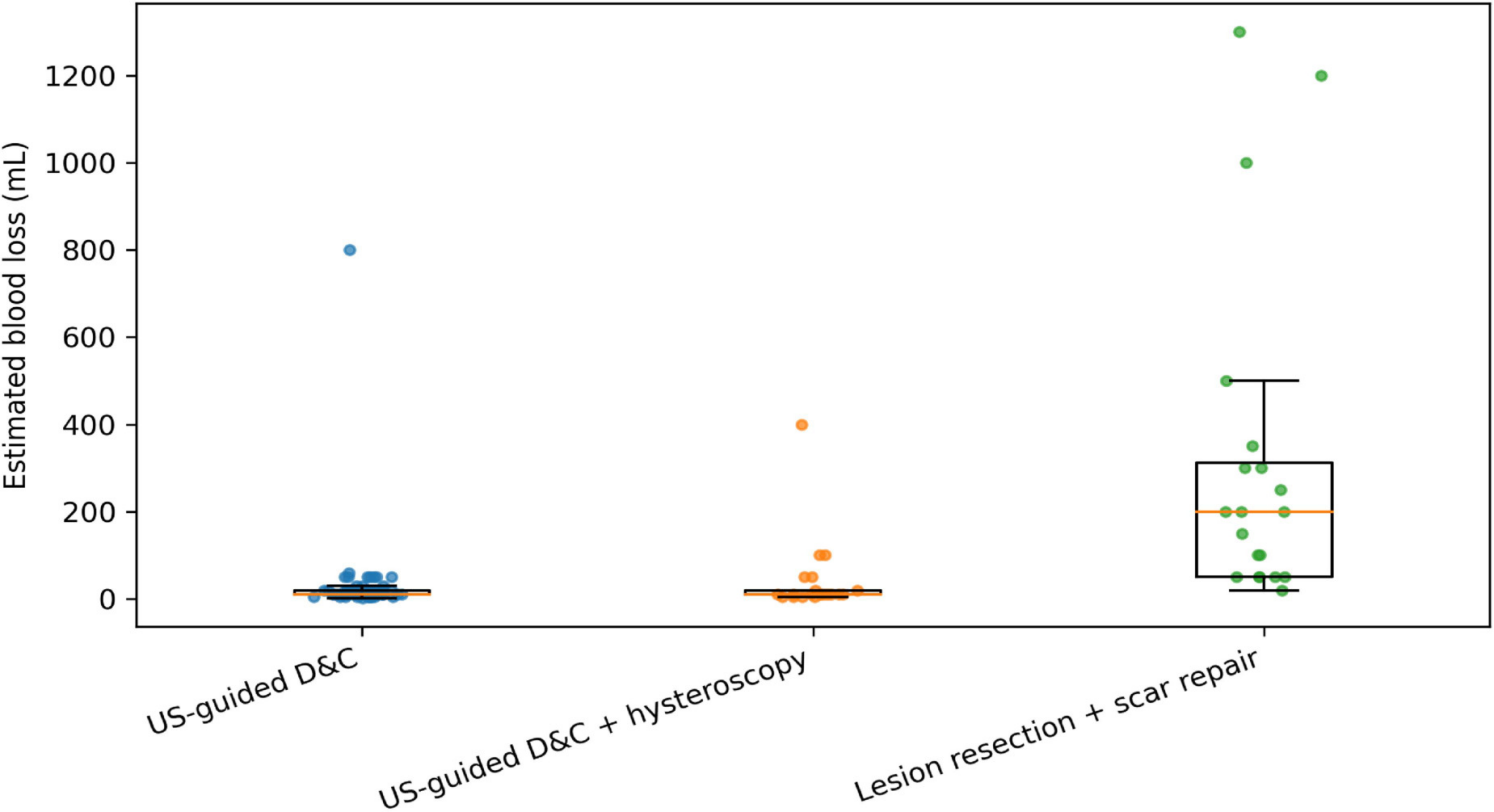
Estimated blood loss by definitive procedure.

**Figure 5:**
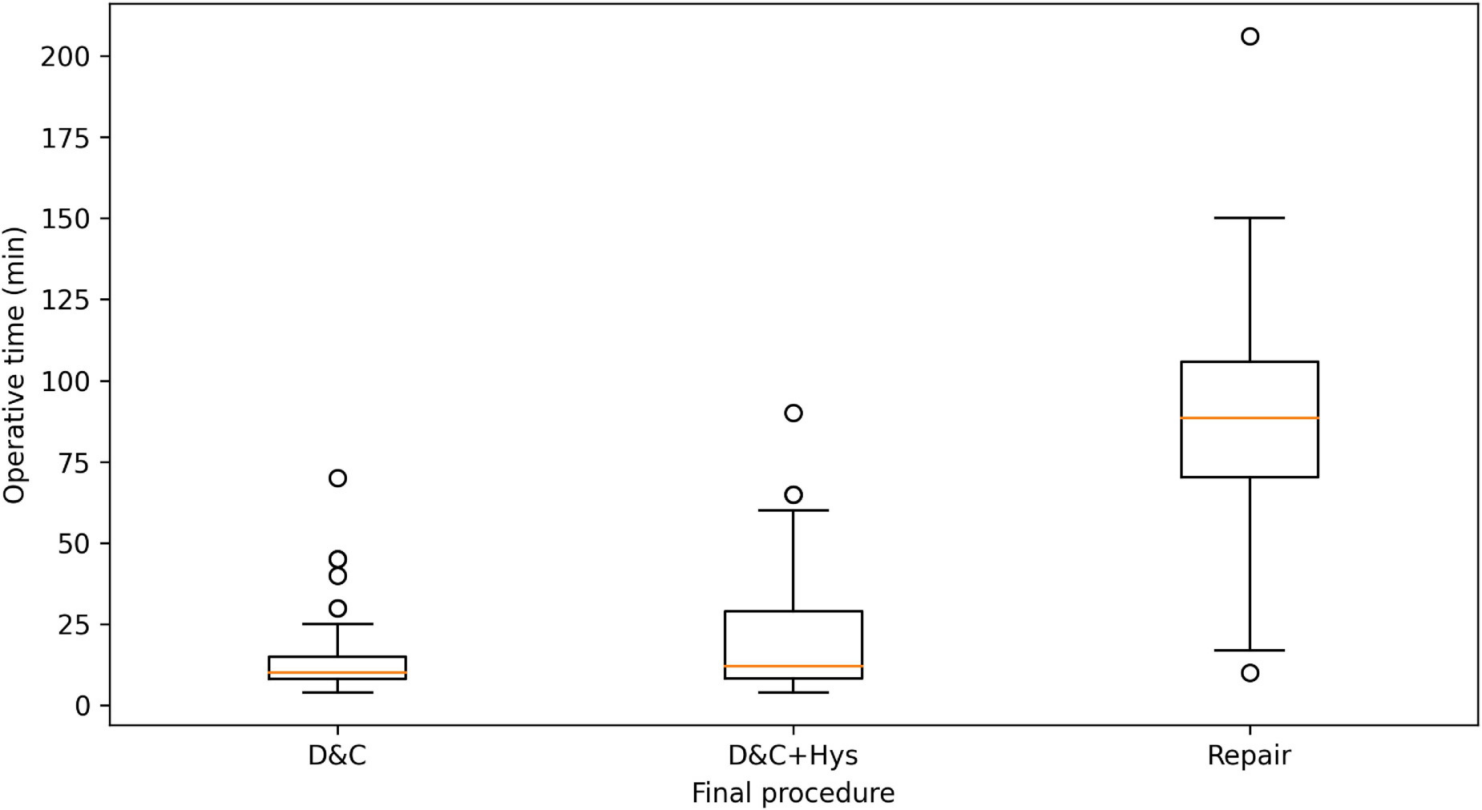
Operative time by definitive procedure.

Operative time was available in 130/131 patients, estimated blood loss (EBL) in 120/131, and length of stay (LOS) in all 131. Because definitive procedure selection reflected baseline lesion severity and operative complexity, comparisons across procedure groups should be interpreted as descriptive and susceptible to confounding by indication. Overall, the median operative time was 12.0 min (IQR 8.0–25.0), median EBL was 12.5 mL (IQR 10.0–50.0), and median LOS was 4.0 days (IQR 3.0–5.0). Perioperative outcomes differed significantly by definitive procedure, with lesion resection plus scar repair associated with longer operative time, greater blood loss, and longer LOS than less invasive approaches (all *P* < 0.001; Table 4; Figures 4, 5).

**Table 4:** Perioperative outcomes by definitive procedure.

Outcome	US-guided D&C	US-guided D&C + hysteroscopy	Lesion resection+scar repair	*P* value
N	84	26	21	—
Operative time, min	10.0 (8.0–15.0) (*n* = 84)	12.0 (8.2–29.0) (*n* = 26)	88.5 (70.2–105.8) (*n* = 20)	<0.001
Estimated blood loss, mL	10.0 (10.0–20.0) (*n* = 79)	10.0 (10.0–20.0) (*n* = 21)	200.0 (50.0–312.5) (*n* = 20)	<0.001
Length of stay, days	3.0 (3.0–4.0) (*n* = 84)	4.0 (3.0–6.0) (*n* = 26)	7.0 (5.0–9.0) (*n* = 21)	<0.001
Any clinical adverse event	1/84 (1.2%)	1/26 (3.8%)	3/21 (14.3%)	0.020
Blood loss >200 mL (among cases with EBL recorded)	1/79 (1.3%)	1/21 (4.8%)	8/20 (40.0%)	<0.001

Continuous variables are median (IQR) with available-case *n* in parentheses. *P* values are from Kruskal–Wallis (continuous) or chi-square (categorical) tests.

Clinical adverse events were recorded in 5/131 (3.8%) patients, occurring more frequently after lesion resection plus scar repair (3/21, 14.3%) than after D&C (1/84, 1.2%) or D&C plus hysteroscopy (1/26, 3.8%) (chi-square *P* = 0.020). Using an EBL threshold of >200 mL among cases with EBL recorded, significant bleeding occurred in 10/120 (8.3%) cases, predominantly in the lesion resection plus scar repair group (8/20, 40.0%).

### 3.5 Early postoperative *β*-hCG kinetics (available-case analysis)

Early postoperative beta-hCG data (earliest available measurement) were available in 71/131 (54.2%) patients. The first postoperative beta-hCG was measured at a median of 1 day after the procedure (IQR 1–2). Across available cases, the median decline from the preoperative reference value to the earliest postoperative value was substantial, but interpretation is limited because postoperative sampling was not standardized and serial follow-up measurements were unavailable for many patients. Accordingly, these findings are presented descriptively as available-case information rather than as a definitive comparative efficacy endpoint (Table 5).

**Table 5:** Early postoperative *β*-hCG kinetics (available cases).

Group	n with early postop *β*-hCG	Postop day of first *β*-hCG, median (IQR)	*β*-hCG decline (%), median (IQR)
Overall	71	1 (1–2)	70.6 (52.8–81.6)
US-guided D&C	46	1 (1–2)	72.0 (61.3–86.7)
US-guided D&C + hysteroscopy	16	1 (1–1)	55.8 (8.1–63.1)
Lesion resection+scar repair	9	1 (1–4)	76.2 (72.7–95.8)

β-hCG decline is reported as percent change from the preoperative reference to the earliest postoperative value; follow-up β-hCG was not systematically available for all patients.

### 3.6 Incremental value of CEUS for predicting complex surgery

Lesion resection plus scar repair was performed in 21/131 (16.0%) patients. The CEUS CCSPB pattern was strongly associated with scar repair (19/20, 95.0%) compared with other patterns (2/111, 1.8%). In multivariable logistic regression, adding a CEUS high-risk indicator (CCSPB) to a baseline TVS-based model significantly improved model fit (likelihood ratio *χ*^2^ = 23.62, df = 1, *P* < 0.001), reduced −2 log likelihood from 46.61 to 22.99, and increased Nagelkerke R^2^ from 0.697 to 0.864. Discrimination improved from AUC 0.949 to 0.989. In the adjusted model, CCSPB was independently associated with scar repair (aOR 318.6, 95% CI 12.5–8,096.8; *P* < 0.001). Using CCSPB alone to triage scar repair yielded sensitivity 90.5% and specificity 99.1%. The full multivariable logistic regression results are presented in Table 6.

**Table 6:** Multivariable logistic regression for predicting lesion resection plus scar repair.

Predictor	Adjusted OR (95% CI)	*P* value
TVS type III (vs. type I/II)	2.16 (0.08–59.82)	0.650
Residual myometrial thickness ≤3 mm	7.23 (0.16–326.08)	0.309
Max gestational sac diameter (per 1 mm)	1.08 (0.97–1.21)	0.153
Viable embryo present	0.47 (0.03–8.98)	0.619
CEUS high-risk pattern (CCSPB)	318.64 (12.54–8,096.78)	<0.001

Baseline covariates were TVS high-risk type (Type III vs. Type I/II), RMT ≤3 mm, maximum sac diameter, and embryo viability; CEUS high-risk pattern was defined as CCSPB. All models were estimated with enter method. AUC, area under the ROC curve.

## 4 Discussion

In this retrospective cohort of 131 cesarean scar pregnancy cases, we developed and implemented a pragmatic contrast-enhanced ultrasound (CEUS) enhancement-pattern classification intended for real-world procedure selection and perioperative risk stratification. CEUS can provide real-time information on microvascular perfusion and the spatial extent of scar-associated trophoblastic vascularity that is not fully captured by grayscale ultrasound alone (12–16). Within our CEUS-evaluated cohort, the proposed classification tracked closely with established severity markers, showed high concordance between recommended and definitive procedures, and identified a high-risk pattern (CCSPB) that added discriminatory information beyond selected TVS variables for cases ultimately managed with scar repair.

Our findings are consistent with contemporary CSP risk-stratification concepts that prioritize implantation phenotype and myometrial integrity when planning treatment. International consensus work has emphasized standardized reporting of key imaging domains such as gestational sac position relative to the scar niche, residual myometrial thickness, implantation depth, and the crossover sign (5, 6). More recent clinical classification systems have further linked these sonographic features to recommended surgical strategies (8, 9). Rather than replacing these established frameworks, our CEUS scheme should be viewed as a perfusion-oriented complement: it captures the distribution and intensity of enhancement within the implantation bed, thereby providing additional information on vascular extension across the scar thickness and toward the serosal/bladder side. This conceptual positioning may explain why the CEUS categories aligned with TVS type, RMT, and scar thickness while still offering incremental information for real-world procedure selection.

CEUS-specific evidence in CSP remains limited and has predominantly focused on diagnostic characterization rather than explicit decision pathways for procedure selection and perioperative outcome prediction. A recent systematic review identified only a small number of eligible CEUS studies, most centered on diagnosis rather than treatment selection (16). Against this background, our study extends the literature by operationalizing CEUS morphology into a procedure-oriented enhancement-pattern framework. At the same time, our findings should be interpreted within the broader management literature, in which treatment selection remains multifactorial and is influenced by lesion morphology, myometrial thickness, vascularity, bleeding risk, reproductive goals, and local surgical expertise (8–11). Recent post-2020 reports have also continued to refine suction-curettage-based and combined surgical strategies in selected CSP cases, underscoring the importance of matching imaging phenotype with procedural burden rather than assuming one universally optimal approach (17–19).

Several limitations should be considered when interpreting these findings. First, this was a retrospective single-center study conducted within a specific inpatient workflow in which CEUS was routinely used after admission for suspected CSP; accordingly, external generalizability to centers with different referral pathways, admission thresholds, or imaging algorithms remains limited. Second, although two experienced readers showed substantial agreement after standardized training (*κ* = 0.775 for the 2016 TVS classification and *κ* = 0.800 for the CEUS enhancement-pattern classification), the retrospective design did not ensure complete blinding to clinical context, so some observer bias may still have persisted. Third, not all contemporary CSP descriptors - such as growth direction, crossover sign, and formal vascularity grading - were uniformly archived in the retrospective dataset, limiting direct comparison with every existing stratification framework. Fourth, postoperative beta-hCG follow-up was incomplete and non-standardized, so those results are descriptive only. Fifth, the regression model for scar repair was based on a limited number of events, and the very large odds ratio observed for CCSPB with wide confidence intervals likely reflects sparse-data instability; no formal calibration or internal validation analysis was available. Finally, the worse perioperative outcomes observed in the scar-repair group should not be interpreted causally, because patients selected for more complex surgery inherently represented higher-risk lesions, introducing confounding by indication.

Future work should prospectively validate this enhancement-pattern classification in multicenter cohorts with standardized acquisition and interpretation protocols, incorporate prespecified blinded rereading procedures, and evaluate CEUS-guided decision pathways against conventional ultrasound-based strategies using prespecified endpoints such as major hemorrhage, rescue interventions, re-intervention, time to resolution, and patient-centered recovery measures. Formal integration of CEUS features with established CSP markers - such as growth direction, crossover sign, residual myometrial thickness, and vascularity grading - would help determine whether perfusion-based classification offers reproducible incremental value beyond current sonographic frameworks.

## 5 Conclusion

CEUS enhancement-pattern classification was closely aligned with conventional ultrasound severity markers and was associated with high concordance between recommended and definitive procedures in cesarean scar pregnancy. A CEUS high-risk pattern (CCSPB) may provide incremental value beyond selected TVS parameters for identifying patients ultimately managed with scar repair, but this signal requires cautious interpretation and external validation. Prospective multicenter studies are warranted to standardize interpretation and determine whether CEUS-guided stratification improves clinically relevant outcomes.

## Data Availability

The raw data supporting the conclusions of this article will be made available by the authors, without undue reservation.
